# Gender Differences in the Interest in Mathematics Schoolwork Across 50 Countries

**DOI:** 10.3389/fpsyg.2020.578092

**Published:** 2020-11-25

**Authors:** Kimmo Eriksson

**Affiliations:** ^1^School of Education, Culture and Communication, Mälardalen University, Västerås, Sweden; ^2^Centre for Cultural Evolution, Stockholm University, Stockholm, Sweden

**Keywords:** learning attitudes, gender differences, mathematics achievement, peer influence, female amplification

## Abstract

Although much research has found girls to be less interested in mathematics than boys are, there are many countries in which the opposite holds. I hypothesize that variation in gender differences in interest are driven by a complex process in which national culture promoting high math achievement drives down interest in math schoolwork, with the effect being amplified among girls due to their higher conformity to peer influence. Predictions from this theory were tested in a study of data on more than 500,000 grade 8 students in 50 countries from the 2011 and 2015 waves of TIMSS. Consistent with predictions, national achievement levels were strongly negatively correlated with national levels of math schoolwork interest and this variation was larger among girls: girls in low-achievement, high-interest countries had especially high interest in math schoolwork, whereas girls in high-achievement, low-interest countries had especially low interest in math schoolwork. Gender differences in math schoolwork interest were also found to be related to gender differences in math achievement, emphasizing the importance of understanding them better.

## Introduction

Children and young adolescents are typically obliged to go to school and must take part in schoolwork even if they do not find it interesting. Nonetheless, it is preferable that students are interested in their schoolwork, both because they are likely to experience more satisfaction in school and because they are likely to achieve better (e.g., Artelt et al., 2003). Given these benefits of having high levels of interest, it is problematic that a large body of research has found that girls tend to have less interest in mathematics than boys do (Hyde et al., 1990; Lippa, 1998; Preckel et al., 2008; Su et al., 2009; Frenzel et al., 2010). However, this gender gap in mathematics interest does not seem to be universal. Recent research using cross-national data from the Trends in Mathematics and Science Survey (TIMSS) has uncovered that in many countries the gender gap in mathematics attitudes, including interest in schoolwork, is reversed (Ghasemi and Burley, 2019; Reilly et al., 2019). These findings suggest that the correct question to ask is not why girls are less interested in math than boys are, because often the opposite holds. From an egalitarian perspective, however, a gender gap in interest in a key subject in school may be regarded as equally problematic regardless of whether it favors boys or girls. Therefore, the present research aims to better understand why the level of interest in mathematics schoolwork may differ between the average boys and the average girls in a society develop, and why the difference may go either way depending on the society.

The theoretical idea I propose is that gender differences in interest in schoolwork may be influenced by a societal factor—the achievement culture, which tends to drive interest in schoolwork down—in combination with a gender difference in conformity, with girls tending to conform more than boys. The outcome, I argue, would be a specific, complex pattern. In high-achievement cultures, it would be common for students to have a low level of interest in math schoolwork and, due to conformity, a low level of interest would be especially common among girls. In low-achievement cultures, by contrast, it would be common for students to have a high level of interest in math schoolwork and, again due to conformity, a high level of interest would be especially common among girls. Thus, high-achievement cultures would exhibit gender gaps in math schoolwork interest that favor boys, while gender gaps would be reversed in low-achievement cultures. Below I develop this novel hypothesis in greater detail, grounding its assumptions in previous literature.

### The Impact of High-Achievement Culture on Students’ Math Schoolwork Interest

**FIGURE 1:**
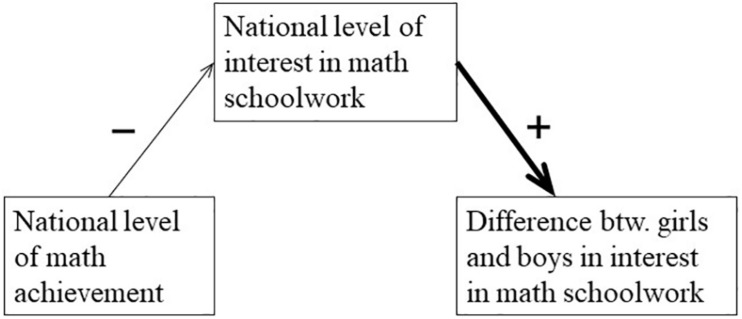
The hypothesized path from high-achievement culture to gender differences in interest in math schoolwork. The first arrow represents the hypothesis that high-achievement nations will have lower average student interest in math schoolwork. The second (bold) arrow represents the hypothesis that differences in average national interest will be amplified among girls.

The achievement culture of a society may be an important factor behind how interested students are in mathematics schoolwork. When comparing across countries, it is well-known that a high average level of student achievement in mathematics and science is related to a range of negative outcomes, including more negative attitudes to math and science (Artelt et al., 2003; Shen and Tam, 2008; Leung, 2014; Täht et al., 2014), worse student-teacher relations (Mikk et al., 2016), and worse academic self-concept among students (Shen and Tam, 2008; Van de gaer et al., 2012; Leung, 2014). For instance, on a global scale, math achievement is very low in Egypt and very high in Japan; nonetheless, a high positive affect toward mathematics was found to be very common among grade 8 students in Egypt but very uncommon in the same grade in Japan (see Table 21 in Leung, 2014). This phenomenon has been described as “paradoxical” (Shen and Tam, 2008). At the individual level, positive attitudes, good student-teacher relations, and a positive academic self-concept are generally regarded as conducive to learning (Artelt et al., 2003). Yet, a national culture that focuses on high achievement may bring about more negative attitudes, worse student-teacher relations, and a more negative academic self-concept. To explain this phenomenon, researchers have pointed to particularly high educational norms and standards in high-achievement countries (Van de gaer et al., 2012). Many students may struggle to fully meet these high standards, even though their achievement is high on a global scale, and this could account for the surprisingly low academic self-concept in high-achievement countries (Van de gaer et al., 2012). The level of interest in schoolwork could similarly be driven down by high educational norms and standards. Students who are struggling to keep up with a progressively difficult curriculum in math could lose interest in doing the schoolwork to progress to even more advanced math, instead preferring to consolidate their knowledge. Consistent with this body of research, I therefore expect that a higher average level of math achievement in a society is linked to a lower average level of interest in math schoolwork. The first (fine) arrow in the diagram in Figure 1 illustrates this hypothesized negative relation.

### Peer Influence Among Girls and Boys

It is well-known that students’ motivation may be heavily influenced by their peers, both positively and negatively, and both intentionally and unintentionally (Kindermann, 2016; Wentzel and Muenks, 2016). Thus, if high-achievement culture makes some students lose interest in math schoolwork, it may negatively influence their peers’ level of interest as well. Moreover, this effect of peer influence could differ between boys and girls. Studies of peer influence in school that have addressed the gender aspect have found that, compared to boys, girls’ interest in schoolwork is more susceptible to peer influence (Berndt and Keefe, 1995; Riegle-Crumb et al., 2006). This effect has been attributed to girls’ friendships being more supportive and discussion-oriented, compared to boys’ friendships that tend to be more competitive and center more on specific activities (Riegle-Crumb et al., 2006; see also Beutel and Marini, 1995). Gender differences in the susceptibility to peer influence in school settings are consistent with findings from conformity research is general. Several meta-analyses have found conformity to be stronger among women than among men (Eagly and Carli, 1981; Bond and Smith, 1996). Although most of these studies have been carried out in Western countries, stronger conformity among women than among men was also observed in a recent study in Sudan when participants evaluated appropriate behavior (Efferson and Vogt, 2018). The gender effect on conformity has been explained in terms of women having less confidence and being more risk-averse than men (Cross et al., 2017; Brand et al., 2018).

Based on this previous literature I expect that within-society conformity with respect to interest in math schoolwork will be more accentuated among women than among men (through mechanisms such as gender differences in confidence, risk aversion, and friendships). However, the focus of the present research is on *between-society* variation, and greater conformity within societies is likely to lead to larger variation between societies. To understand this theoretical point, consider boys and girls in a high-achievement culture where some students lose their interest in math schoolwork due to high educational norms and standards. To some degree they will exert a negative influence on their peers’ interest, and this peer influence is expected to be more pervasive among girls than among boys. Thus, the direct negative effect of high-achievement culture on students’ interest is expected to be amplified by social dynamics, and this amplification is expected to be stronger among girls than among boys. The result would be greater variation across societies in the average interest levels of girls than in the average interest levels of boys.

This hypothesis is illustrated in the diagram in Figure 1, where the second (bold) arrow signifies the gender-specific amplification of national differences in interest in math schoolwork. Together, the two arrows describe a hypothetical indirect negative effect of a high national level of math achievement on gender differences in math schoolwork interest, mediated by the national level of math schoolwork interest.

### The Impact of Gender Differences in Math Schoolwork Interest on Math Achievement

Gender differences in math schoolwork interest are important not least because they are likely to impact on the math achievement of boys and girls. At the individual level, interest in math schoolwork is thought to be conducive to learning (Artelt et al., 2003). In societies where girls tend to be less interested in math schoolwork than the boys are, it could contribute to a corresponding gender difference in math achievement. Why the gender gap in math achievement varies across societies has been the topic of extensive research for decades (e.g., Guiso et al., 2008; Else-Quest et al., 2010). A recent study, using data from all waves of the TIMSS and PISA assessments between 2000 and 2015, found the societal level of gender egalitarian values to be the strongest and most robust predictor of gender differences in achievement in math, science, and reading, but there was still a large amount of unexplained variation (Eriksson et al., 2020). I hypothesize that some of this variation is accounted for by gender differences in interest in schoolwork. This would underscore the importance of the study’s main aim of understanding why these gender differences vary across countries.

### Research Questions

Above I have outlined a theory about antecedents and consequences of societal levels of math schoolwork interest among girls and boys. To fully test claims of causality would require experimental or, at least, longitudinal data, neither of which are available. Instead, I here make do with analyzing cross-sectional data provided by TIMSS. The theory predicts certain statistical patterns to arise in such data and the aim of the empirical part of this study is to examine whether these patterns can indeed be observed. It is an important first test of the theory to see whether it correctly predicts several non-trivial features of a complex dataset, even though alternative causal accounts cannot be excluded.

RQ1. The hypothesis of high-achievement culture impacting on students’ math schoolwork interest yields the first prediction to be examined: Is there a negative correlation between national levels of achievement and math schoolwork interest?

RQ2. The hypothesis of a difference between boys and girls in peer influence on math schoolwork interest yields a suite of testable predictions: (a) Is within-society variation in math schoolwork interest smaller among girls than among boys? (b) Is between-society variation in math schoolwork interest larger among girls than among boys? (c) Is there a positive correlation between national levels of math schoolwork interest and gender gaps in math schoolwork interest favoring girls? (d) Do national levels of math schoolwork interest mediate a negative correlation between national levels of achievement and gender gaps in math schoolwork interest favoring girls?

RQ3. The hypothesis that gender differences in math schoolwork interest has an independent impact on the gender gap in math achievement also yields a testable prediction: Does the gender gap in math schoolwork interest account for some of the variance in the gender gap in math achievement, over and beyond the variation already accounted for by gender egalitarian values?

## Materials and Methods

To answer the research questions, the current study analyzes TIMSS data. TIMSS is an excellent resource for comparative research as it uses large representative national samples of students from many countries. Details on the design are provided by the International Association for the Evaluation of Educational Achievement (Martin et al., 2016). In brief, TIMSS assesses math achievement of students in the eighth grade, in which most participants are about 14 years old. In addition to the achievement test, participating students also complete a background questionnaire. This questionnaire is not fixed across waves. In the 2011 and 2015 waves of TIMSS, the questionnaire included items on students’ interest in what the teacher says and students’ interest in what the teacher tells them to do. No such questions were included in previous waves of TIMSS, nor have they been included in other large-scale international student assessments like PISA. For this reason, this study will use data from the 2011 and 2015 waves of TIMSS.

Data from the 2011 and 2015 waves of TIMSS were downloaded from IEA^1^. Data were available for a total of 50 countries, out of which 35 countries had participated in both waves, 10 countries had participated only in the 2011 wave, and 5 countries only in the 2015 wave. See Table 1 for countries and samples sizes in each wave. All populated world continents were represented, including 24 countries in Asia from Israel and Saudi Arabia in the west to Korea and Japan in the east, 6 countries in Africa from Morocco and Egypt in the north to Botswana and South Africa in the south, 4 countries in the Americas from Canada to Chile, 14 countries in Europe from Sweden to Malta, as well as Australia and New Zealand.

**TABLE 1:** TIMSS sample sizes and key measures.

Country	2011	2015	Math schoolwork interest				Mean math		Gender
	Sample size	Sample size	Girls		Boys		Achievement		Egalitarian values
			M	SD	M	SD	Girls	Boys	
Armenia	7,556	10,338	3.52	0.63	3.45	0.69	472.8	464.8	−0.91
Australia	4,640	4,918	2.70	0.79	2.79	0.78	502.6	508.2	1.09
Bahrain	5,846	5,060	3.08	0.76	3.05	0.84	446.4	417.3	
Botswana	5,400	5,964	3.36	0.68	3.31	0.71	401.8	385.4	
Canada		8,757	2.97	0.74	3.01	0.74	525.8	530.8	1.31
Chile	5,835	4,849	3.07	0.78	3.12	0.79	414.0	430.1	0.31
Chinese Taipei	5,042	5,711	2.41	0.74	2.51	0.81	605.8	602.7	
Egypt		4,035	3.50	0.69	3.43	0.74	396.6	387.4	−1.87
Finland	4,266		2.42	0.72	2.40	0.73	516.6	512.1	0.62
Georgia	4,563	4,155	3.39	0.65	3.33	0.70	441.7	442.7	−0.85
Ghana	7,812		3.53	0.58	3.58	0.55	318.7	342.2	−0.65
Honduras	7,323		3.53	0.65	3.51	0.68	327.8	351.0	
Hong Kong	4,418	4,893	2.58	0.75	2.73	0.81	590.2	590.0	
Hungary	4,015	6,130	2.74	0.77	2.74	0.81	505.8	513.3	0.52
Indonesia	5,178		3.14	0.44	3.10	0.48	392.4	379.5	−0.56
Iran	5,795	4,704	3.16	0.75	3.24	0.76	424.6	426.5	−1.10
Ireland		5,512	2.75	0.81	2.80	0.82	520.8	526.3	1.43
Israel	6,029	4,481	2.91	0.82	2.88	0.85	515.1	512.6	0.68
Italy	4,699	4,745	2.82	0.70	2.85	0.74	491.9	500.5	0.87
Japan	3,979	4,887	2.26	0.66	2.39	0.72	576.8	578.4	−0.13
Jordan	4,414	7,865	3.47	0.65	3.43	0.71	407.6	384.3	−1.55
Kazakhstan	4,390	5,309	3.42	0.55	3.31	0.58	508.6	506.2	0.13
Korea	7,694	4,503	2.28	0.65	2.38	0.71	607.4	611.3	−0.23
Kuwait		3,873	3.01	0.79	3.23	0.76	396.0	388.7	−1.39
Lebanon	5,166	4,347	3.30	0.76	3.30	0.76	442.3	449.9	−0.32
Lithuania	3,974	9,726	2.91	0.73	2.92	0.75	508.5	505.3	−0.09
Macedonia	4,747		3.23	0.79	3.23	0.78	429.6	422.6	0.09
Malaysia	5,733	3,817	3.18	0.63	3.04	0.67	459.5	445.5	−0.98
Malta		13,035	2.80	0.81	2.93	0.83	495.1	492.7	
Morocco	8,986	8,883	3.49	0.64	3.46	0.66	378.5	377.8	−0.97
New Zealand	9,542	8,142	2.72	0.78	2.85	0.77	486.8	494.6	0.49
Norway	5,336	4,697	2.70	0.77	2.78	0.78	493.6	493.1	1.90
Oman	3,862	5,403	3.52	0.56	3.31	0.71	408.4	361.0	
Palestine	4,422		3.57	0.57	3.38	0.72	415.3	392.2	
Qatar	5,523	4,780	3.01	0.81	3.08	0.83	427.8	419.1	−1.48
Romania	4,893		3.05	0.79	2.98	0.81	463.7	452.8	−0.13
Russia	4,344	3,759	3.09	0.69	3.09	0.70	535.9	541.0	−0.30
Saudi Arabia	5,927	6,116	3.21	0.74	3.16	0.81	388.1	373.5	−1.79
Singapore	4,415	4,257	2.89	0.68	2.94	0.72	620.5	611.7	0.20
Slovenia	11,969	12,514	2.55	0.69	2.55	0.75	508.8	512.4	0.86
South Africa	5,573	4,090	3.41	0.65	3.38	0.68	364.8	359.5	0.04
Sweden	4,413	6,482	2.61	0.71	2.73	0.74	492.5	493.8	1.66
Syria	6,124		3.55	0.60	3.48	0.69	374.9	384.7	
Thailand	14,089	18,012	3.29	0.55	3.24	0.60	437.3	419.7	−0.20
Tunisia	5,128		3.47	0.65	3.43	0.68	416.8	433.6	
Turkey	6,928	6,079	3.11	0.65	3.06	0.71	459.0	451.6	−0.17
UAE	3,378	7,822	3.15	0.73	3.15	0.77	467.7	453.1	
United Kingdom	4,062	10,221	2.71	0.76	2.83	0.77	514.7	511.5	1.25
Ukraine	10,477		3.30	0.66	3.27	0.69	477.6	481.0	−0.25
United States	3,842	4,814	2.80	0.83	2.83	0.83	512.9	515.5	1.18

*All measures are based on TIMSS data except the measure of gender egalitarian values, which is based on data from World Values Survey and GLOBE.*

The TIMSS datasets come with appropriate sampling weights, which were used when calculating the below measures. Missing data (less than 3% of data) were ignored. Preliminary analyses revealed that country measures were highly consistent across the two waves. For the below analysis we therefore pooled the individual data from the two waves.

### Students’ Interest in Math Schoolwork

The student questionnaire in the 2011 and 2015 waves of TIMSS included three items bearing explicitly on interest in math schoolwork: “I am interested in what my teacher says,” “My teacher gives me interesting things to do,” and “I learn many interesting things in mathematics.” For each item, students gave their response on a four-point scale: *Disagree a lot* (coded 1), *Disagree a little* (coded 2), *Agree a little* (coded 3), *Agree a lot* (coded 4). These three items were averaged to an internally consistent measure of students’ interest in math schoolwork (α = 0.78). Mean value and standard deviation, separately among girls and among boys, are reported per country in Table 1.

### Student Achievement in Mathematics

TIMSS provides ready calculated national average scores for girls’ and boys’ math achievement, which were downloaded using the International Data Explorer of the National Center for Education Statistics^2^ (see Table 1).

### Gender Egalitarian Values

Following Eriksson et al. (2020), gender egalitarian values were measured using the Equality index from the World Values Survey (Welzel, 2013) and the Gender Egalitarian Cultural Values index from the GLOBE project (House et al., 2004). Both measures are based on survey responses to items on how society should be with respect to gender equality in education, leadership, and jobs. From Eriksson et al. (2020), measures of gender egalitarian values were obtained for 39 out of the 50 countries in the study: 26 countries had measures from both WVS and GLOBE, 11 countries only from WVS, and 2 countries only from GLOBE. On the set of 26 countries for which both measures were available, they were very strongly correlated, *r* = 0.85, indicating that they indeed measure the same construct so that the measures can be combined. After transforming both WVS and GLOBE measures to z-scores (i.e., standardizing both measures to have the same mean value, zero, and the same standard deviation, one), I combined them into a single measure, using their average for any country where both measures were available.

## Results

Country levels of math schoolwork interest, general affect toward math, and math achievement, were calculated as the averages of the corresponding mean values for girls and boys. Gender differences for the same variables were similarly calculated as the mean value for girls minus the mean value for boys. Table 2 presents descriptive statistics of these national levels and gender differences.

**TABLE 2:** Descriptive statistics for country levels and gender differences of the measures in Table 1.

Variable	*M*	*SD*	Min	Max
Country level of math schoolwork interest	3.06	0.34	2.33	3.55
Country level of math achievement	464.3	70.3	330	616
Gender difference in math schoolwork interest	−0.01	0.09	−0.22	0.20
Gender difference in math achievement	2.9	12.7	−24	47

*Based on n = 50 countries. Country levels are calculated as the average of the mean values for girls and boys in Table 1, while gender differences are calculated as the girls’ mean value minus the boys’ mean value.*

### RQ1: The Predicted Relation Between Country Levels of Achievement and Math Schoolwork Interest

The first research question concerns the prediction of a negative correlation between country levels of achievement and math schoolwork interest. In line with the prediction, a very strong negative correlation was observed, *r*(48) = −0.81, 95% CI [−0.70, −0.89], *p* < 0.001. Here and throughout, I report bias corrected accelerated confidence intervals based on 1,000 bootstrap samples generated by SPSS v. 26.

### RQ2: Predictions Based on an Assumed Difference in Peer Influence on Girls’ vs. Boys’ Math Schoolwork Interest

#### RQ2a: Is within-society variation in math schoolwork interest smaller among girls than among boys?

In Table 1, the standard deviation in math schoolwork interest was smaller among girls than among boys in 43 out of 50 countries, in line with the prediction. After transforming standard deviations to variances, the mean difference between girls (*M* = 0.497, *SD* = 0.115) and boys (*M* = 0.545, *SD* = 0.104) was −0.048, 95% CI [−0.062, −0.034], *t*(49) = −6.93, *p* < 0.001, *d* = 0.98, paired samples *t*-test.

#### RQ2b: Is Between-Society Variation in Math Schoolwork Interest Larger Among Girls Than Among Boys?

**FIGURE 2:**
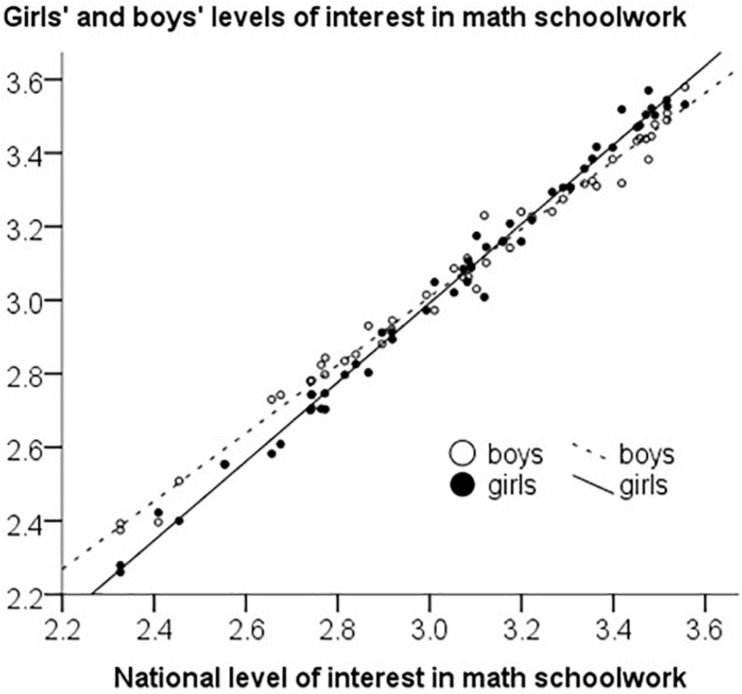
Country variation in math schoolwork interest is amplified among girls. In high-interest countries, the interest levels of girls (black dots, solid line) tend to be higher than the interest levels of boys (circles, dashed regression line), while in low-interest countries girls tend to have lower interest levels than boys do.

Figure 2 presents a scatterplot of girls’ and boys’ levels of interest in math schoolwork plotted against the average level. As indicated by the regression lines, girls in high-interest countries are even more interested than the boys are, while girls in low-interest countries are even less interested than the boys. Thus, between-society variation was larger for girls, in line with the prediction. To quantify the difference, the country variance of the math schoolwork interest level among girls was σ^2^ = 0.133, which is 36% higher than the corresponding country variance among boys, σ^2^ = 0.098. To estimate the statistical significance, we use the Morgan-Pitman test for difference in variance in paired data. This test assumes normally distributed data, and Kolmogorov-Smirnov tests indicated that the country level data on math schoolwork interest did not deviate from a normal distribution either for girls or boys, *ps* > 0.20. The Morgan-Pitman test says that testing for a difference in variance in paired data is equivalent to testing for a correlation between the mean of the paired variables and the difference between the paired values (Wilcox, 2015). In our case, this means testing for a correlation between the total country level of math schoolwork interest and the gender difference in math schoolwork interest. In other words, research questions RQ2b and RQ2c are statistically equivalent. We conduct the test below.

#### RQ2c: Is There a Positive Correlation Between National Levels of Math Schoolwork Interest and Gender Gaps in Math Schoolwork Interest Favoring Girls?

**FIGURE 3:**
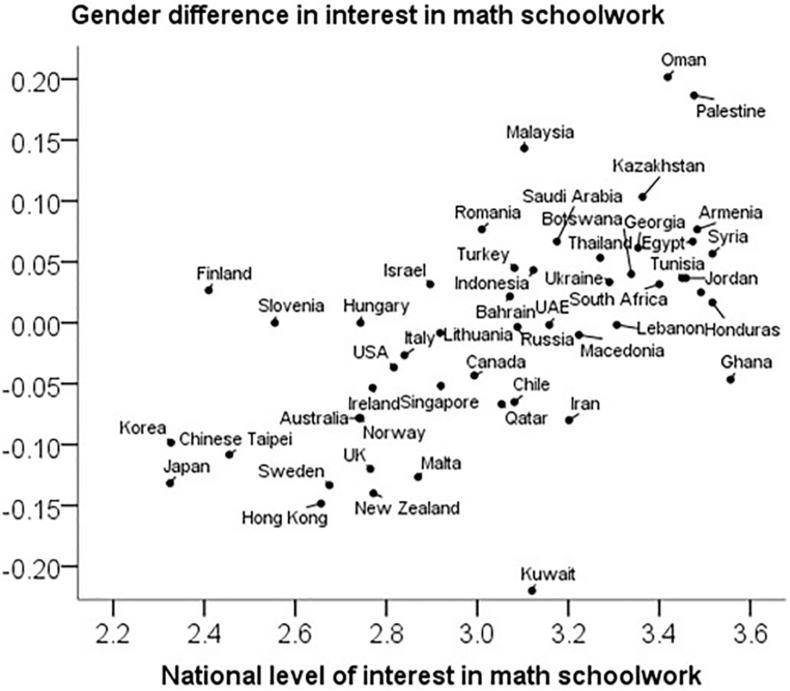
Gender differences (favoring girls) in math schoolwork interest correlate with the national level of interest. The Pearson correlation is *r* = 0.60 (or *r* = 0.66 if the outlier Kuwait is excluded).

In line with the prediction, there was a strong positive correlation between the total country level of math schoolwork interest and the gender difference in math schoolwork interest, *r*(48) = 0.60, 95% CI [0.39, 0.78], *p* < 0.001. However, Kuwait was diagnosed as an outlier (standardized residual > 3) (see Figure 3). If the outlier is excluded, the correlation is even higher, *r*(47) = 0.66.

#### RQ2d: Do National Levels of Math Schoolwork Interest Mediate a Negative Correlation Between National Levels of Achievement and Gender Gaps in Math Schoolwork Interest Favoring Girls?

**FIGURE 4:**
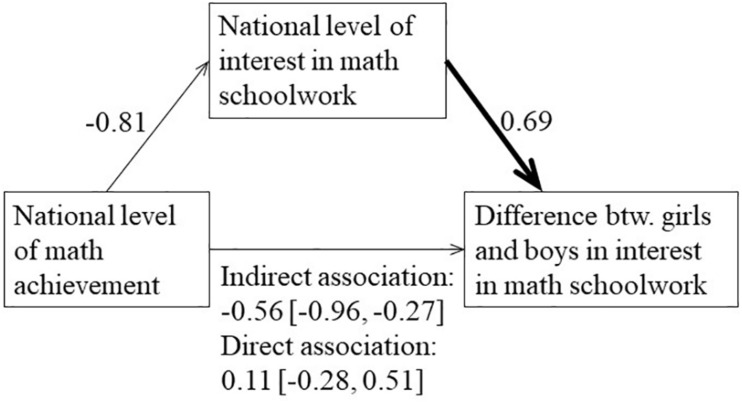
Mediation diagram. The negative correlation between the national math achievement level and the gender difference in math schoolwork interest was mediated by the national level of math schoolwork interest. All variables were standardized prior to the mediation analysis. Bootstrapped 95% confidence intervals in brackets.

In line with the prediction, there was a negative correlation between national levels of math achievement and gender differences in math schoolwork interest, *r*(48) = −0.45, 95% CI [−0.64, −0.22], *p* = 0.001. To examine mediation, I employed the PROCESS macro, model 4, for SPSS (Hayes, 2017), after standardizing all variables to have unit standard deviation. Results are reported in the mediation diagram in Figure 4, showing that the abovementioned correlation was fully mediated by the national interest in math schoolwork.

### RQ3. Does the Gender Gap in Math Schoolwork Interest Account for Variance in the Gender Gap in Math Achievement Unexplained by Gender Egalitarian Values?

There was a positive correlation between gender differences in math schoolwork interest and gender differences in math achievement, *r*(48) = 0.46, 95% CI [0.15, 0.68], *p* < 0.001. Results are virtually unchanged when the analysis is restricted to the subset of 39 countries for which measures of gender egalitarian are available, *r*(37) = 0.41, 95% CI [0.16, 0.63], *p* = 0.001.

Consistent with prior research (Eriksson et al., 2020), gender egalitarian values were negatively correlated gender differences in achievement, *r*(37) = −0.43, 95% CI [−0.66, −0.15], *p* < 0.001. On their own, gender egalitarian values accounted for 19% of the country variation in gender differences in math achievement. In line with the prediction, this proportion increased to 27% when the regression additionally included gender differences in math schoolwork interest, β = 0.31, *p* = 0.049.

## Discussion

The present paper studied the difference between girls and boys in their interest in math schoolwork and how it varies across countries. A theory was proposed according to which national culture promoting high math achievement drives down interest in math schoolwork, but more among girls than among boys due to conformity to peer influence being stronger among girls. Moreover, I argued that gender differences in math schoolwork interest are important because they will contribute to gender differences in math achievement. In the absence of experimental data, I tested the predictions this theory makes about statistical observations in cross-sectional data, provided by TIMSS. Results were consistent with predictions, as detailed below.

First, an extremely strong negative correlation between national levels of achievement and math schoolwork interest was observed. This finding, which is well in line with prior research on the relation between national achievement levels and attitudes to math and science (Shen and Tam, 2008; Täht et al., 2014), is consistent with the hypothesis that students’ interest in schoolwork is negatively influenced by the high educational norms and standards in high-achievement cultures (Van de gaer et al., 2012). That high-achievement culture may be killing students’ interest is arguably a serious problem. Comparisons between high-achieving countries indicate that the problem might be solvable, however. In a study of TIMSS data from 1999 to 2003, Shen and Tam (2008) pointed out that students in Singapore, an extremely high-achieving country, nonetheless had relatively positive attitudes toward math and science. Leung (2002) made the same observation. Singapore was a positive exception also in the current study, having the highest achievement level of all countries in the study, yet exhibiting a much higher national level of interest in math schoolwork than similarly high-achieving Korea and Japan did (Table 1). It would be valuable to understand whether there is some specific feature of Singapore’s school system that mitigates the negative side effects of a high-achievement culture.

Second, several findings were consistent with the hypothesis that conformity to peer influence on math schoolwork interest is higher among girls than among boys. In almost all countries in the study, within-society variation in math schoolwork interest was smaller among girls than among boys, thus indicating greater female conformity. Because societies vary in their average level of interest in math schoolwork, the effect of peer pressure will vary too. Consistent with greater susceptibility to peer influence among girls, between-society variation in math schoolwork interest was larger among girls than among boys (Figure 2): In countries where the interest in math schoolwork was low, it tended to be especially low among girls. Similarly, in countries where the interest in math schoolwork was high, it tended to be especially high among girls. Thus, the country variation in students’ interest in mathematics schoolwork was amplified among girls. The same phenomenon could be observed in terms of a positive correlation between national levels of math schoolwork interest and gender differences in math schoolwork interest favoring girls.

Taken together, my theory proposes a pathway in which high-achievement culture drives down schoolwork interest, which through differential peer influence creates gender gaps in interest disfavoring girls. Consistent with this pathway, I found a negative correlation between national levels of achievement and gender gaps in math schoolwork interest favoring girls, and this correlation was mediated by the national level of math schoolwork interest.

Why is it important how girls’ and boys’ interest in math schoolwork vary across countries? For one thing, it is theoretically important to realize that the variation is substantial. In countries like Japan, Hong Kong, Sweden, and New Zealand, the interest level of the average girl was about 0.2 standard deviations lower than the interest of the average boy. These findings are consistent with research arguing for a fundamental gender difference in subject interest (e.g., Su et al., 2009). But this view appears to be contradicted by the finding of other societies, such as Oman, Malaysia, Palestine, and Kazakhstan, in which the gender gap is at least as wide but reversed.

Gender differences in math interest may also have real-life implications by influencing how girls achieve in mathematics relative to boys in the same country. Consistent with this hypothesis, I found that variation in the gender gap in math schoolwork interest accounts for part of the proportion of variance in the gender gap in math achievement that is not explained by variation in gender egalitarian values (Eriksson et al., 2020).

This study is an example of the benefits of using big data from large-scale assessments of student achievement to examine phenomena in educational psychology. A limitation, inherent in the reliance on cross-sectional data, is that directions of causality are not established. The findings are consistent with the proposed theory, but they could also have arisen from other mechanisms. It is helpful to consider what these alternative mechanisms could be. With respect to the strong negative correlation between national levels of achievement and schoolwork interest, it seems implausible that it would arise from low interest levels having a positive effect on achievement levels. Following Van de gaer et al. (2012), I proposed that high educational norms and standards have lower interest as an undesired side effect. However, there might be something else going on and perhaps more detailed insights into the abovementioned differences between Singapore and its East Asian neighbors could shed more light on this.

Similar reasoning applies to the amplification among girls of national variation in schoolwork interest. I proposed that this arises from differential conformity to peer influence, but it cannot be excluded that there is some alternative societal factor that causes girls’ interest levels to be more extreme than the interest levels of boys. An interesting possibility for future research would be for large-scale assessments to provide some direct measures of peer influence (see also Eriksson et al., 2020).

The idea of conceiving of high-achievement culture as a factor behind gender differences has a precedent. In a study of PISA data, Mann and DiPrete (2016) found that the national achievement level correlated with gender differences in academic self-concept and STEM aspirations. However, they did not examine the female amplification account, that is, whether these effects were mediated by national levels of academic self-concept and STEM aspirations. Future research should examine the scope of female amplification as a mechanism behind gender differences in various beliefs and attitudes.

To conclude, the present study has contributed to scientific understanding of gender differences in interest in mathematics schoolwork by, first, proposing a theory of why such gender differences would arise and vary across countries, and second, testing several theoretical predictions in a large cross-national dataset. Results were consistent with both key components of the theory: high-achievement culture may be detrimental to interest in schoolwork and this effect may be amplified among girls due to their higher conformity to peer influence. These positive findings motivate further study of the validity and scope of the proposed mechanisms.

## Data Availability Statement

Publicly available datasets were analyzed in this study. This data can be found here: https://osf.io/dwk8h/.

## Ethics Statement

Ethical review and approval was not required for the study on human participants in accordance with the local legislation and institutional requirements. Written informed consent from the participants’ legal guardian/next of kin was not required to participate in this study in accordance with the national legislation and the institutional requirements.

## Author Contributions

KE performed the statistical analysis and wrote the manuscript.

## Conflict of Interest

The author declares that the research was conducted in the absence of any commercial or financial relationships that could be construed as a potential conflict of interest.

## Footnotes

1 https://timssandpirls.bc.edu/

2 https://nces.ed.gov/timss/idetimss/
